# Prevalence of alternative lengthening of telomeres in pediatric sarcomas determined by the telomeric DNA C-circle assay

**DOI:** 10.3389/fonc.2024.1399442

**Published:** 2024-08-19

**Authors:** Trevor A. Burrow, Balakrishna Koneru, Shawn J. Macha, Wenyue Sun, Frederic G. Barr, Timothy J. Triche, C. Patrick Reynolds

**Affiliations:** ^1^ Department of Pediatrics, Texas Tech University Health Sciences Center School of Medicine Cancer Center, Lubbock, TX, United States; ^2^ Department of Translational Neuroscience and Pharmacology, Texas Tech University Health Sciences Center, Lubbock, TX, United States; ^3^ Department of Cell Biology and Biochemistry, Texas Tech University Health Sciences Center Graduate School of Biomedical Sciences, Lubbock, TX, United States; ^4^ Laboratory of Pathology, National Cancer Institute, Bethesda, MD, United States; ^5^ Children’s Hospital Los Angles, Department of Pathology and Laboratory Medicine, Keck School of Medicine of University of Southern California, Los Angeles, CA, United States

**Keywords:** rhabdomyosarcoma, Ewing sarcoma, osteosarcoma, alternative lengthening of telomeres, telomere

## Abstract

**Introduction:**

Alternative lengthening of telomeres (ALT) occurs in sarcomas and ALT cancers share common mechanisms of therapy resistance or sensitivity. Telomeric DNA C-circles are self-primed circular telomeric repeats detected with a PCR assay that provide a sensitive and specific biomarker exclusive to ALT cancers. We have previously shown that 23% of high-risk neuroblastomas are of the ALT phenotype. Here, we investigate the frequency of ALT in Ewing’s family sarcoma (EFS), rhabdomyosarcoma (RMS), and osteosarcoma (OS) by analyzing DNA from fresh frozen primary tumor samples utilizing the real-time PCR C-circle Assay (CCA).

**Methods:**

We reviewed prior publications on ALT detection in pediatric sarcomas. DNA was extracted from fresh frozen primary tumors, fluorometrically quantified, C-circles were selectively enriched by isothermal rolling cycle amplification and detected by real-time PCR.

**Results:**

The sample cohort consisted of DNA from 95 EFS, 191 RMS, and 87 OS primary tumors. One EFS and 4 RMS samples were inevaluable. Using C-circle positive (CC+) cutoffs previously defined for high-risk neuroblastoma, we observed 0 of 94 EFS, 5 of 187 RMS, and 62 of 87 OS CC+ tumors.

**Conclusions:**

Utilizing the ALT-specific CCA we observed ALT in 0% of EFS, 2.7% of RMS, and 71% of OS. These data are comparable to prior studies in EFS and OS using less specific ALT markers. The CCA can provide a robust and sensitive means of identifying ALT in sarcomas and has potential as a companion diagnostic for ALT targeted therapeutics.

## 1 Introduction

Telomeres are nucleoprotein structures at the ends of chromosomes (1) that contain 5-10 kilobases of the canonical hexanucleotide (5’-TTAGGG-3’) repeat sequence encased in sheltering proteins (2). This complex protects genomic DNA from replicative erosion (3, 4), shields the ends of chromosomes from aberrant fusion (5), and prevents DNA damage response (DDR) elements from errantly recognizing genomic DNA (2, 6, 7). Approximately 85-90% of all cancers achieve replicative immortality by utilizing the telomere maintenance mechanism (TMM) telomerase (TA), a ribonucleotide reverse transcriptase (8, 9). The remaining 10-15% of cancer cases (~250,000 U.S. patients annually) use a non-telomerase TMM called alternative lengthening of telomeres (ALT) (10, 11).

Incidence of ALT varies amongst sarcomas (**Tables 1**, **2**), with the majority of cases arising from tissues of mesenchymal or neuroepithelial origin (10, 22). Cancers with an estimated ALT frequency >40% include osteosarcoma (OS), diffuse and anaplastic astrocytomas, undifferentiated pleomorphic sarcomas, and pediatric grade 4 glioblastoma multiforme (10, 14). Previously reported patient sample screenings have demonstrated a broad range of ALT frequency amongst pediatric cancers, from 0% in Ewing’s Family Sarcoma (EFS), up to 85% in OS (10, 12, 14). Recently, there have been calls for assessing patient samples with currently available ALT biomarkers to confirm historically reported ALT frequencies, especially for OS (23).

**Table 1:** A review of published pediatric sarcoma data on incidence of ALT-positive tumors.

Histology	Estimated Annual Cases	%ALT	Method	N	Ref
Ewing’s Family Sarcoma	200	0 0 0	UTF TRF, TRAP UTF	23 30 10	(10, 12, 13)
Osteosarcoma	800	66 35 47	TERT, TRAP TERT, TRF, TRAP APB	44 60 58	(14–16)
Rhabdomyosarcoma	350	6 6 0	APB UTF UTF	35 16 4	(10, 13, 14)

**Table 2:** A review of published adult sarcoma data on incidence of ALT-positive tumors.

Histology	Estimated Annual Cases	%ALT	Method	N	Ref
Angiosarcoma	260	24 11 20	UTF UTF UTF	70 9 8	(10, 13, 17)
Chondrosarcoma	1500	100 NA	UTF TRF, APB	2 3	(10, 14)
Leiomyosarcoma	130	78 62 59 53	CCA TRF, APB UTF UTF	49 13 86 59	(10, 14, 18, 19)
Liposarcoma	1500	31 26	UTF TRF, TRAP, APB	75 139	(13, 20)
Myxofibrosarcoma	530	76	UTF	25	(13)
Malignant Peripheral Nerve Sheath Tumor	300	26 21 0	UTF UTF UTF	49 14 4	(10, 13, 21)
Synovial Sarcoma	900	9 0	TRF, APB UTF	11 13	(13, 14)
Undifferentiated Pleomorphic/MFH Sarcoma	2250	77 65 63	TRF, APB UTF UTF	22 34 52	(10, 13, 14, 69)

The hallmarks of the ALT phenotype include absence of TA activity (*TERT* mRNA expression provides a suitable surrogate for TA activity) (24) with the presence of high telomere content and heterogenous telomere length (25, 26), non-canonical telomere variant repeats (27), extra-chromosomal telomeric repeats (28), ALT-associated PML bodies (APBs) (14, 29–31), ultrabright telomere foci by FISH (10), and telomeric DNA C-circles (**Tables 1**, **2**) (32). These characteristic markers have been used to screen tumor sample cohorts to determine the frequency of ALT among various tumor histologies (10, 13, 14, 18, 33, 34). Each of the methods has advantages and disadvantages (**Table 3**).

**Table 3:** Advantages and disadvantages of assays used to determine telomere maintenance mechanisms (TMM).

	TRF	TRAP	TERT	UTF	APB/IF-FISH	Real-time CCA
Advantages	Heterogeneity in telomere length	Direct measure of telomerase activity	High-throughput, Quantitative	Heterogeneity in telomere length, Highly specific, Input/FFPE	Direct ALT measure, Input/FFPE	Direct ALT measure, High-throughput, Plasma monitoring possible, Quantitative, Sensitivity and specificity Widely clinically translatable
Disadvantages	High complexity, Large template input, Sensitivity/specificity, Some TA cells have long telomeres	False negative rate, Indirect ALT measure	Indirect ALT measure, Input/RNA	High complexity, Low throughput	High complexity Low throughput Not all ALT have detectable APBs	CC relatively fragile, Not all ALT have detectable CC

Historically, ALT has been identified by the telomerase repeated amplification protocol (TRAP) assay to demonstrate low TA activity (35) and/or low *TERT* mRNA expression, since TA is mutually exclusive to ALT (36). Telomere content and heterogeneity have been evaluated by telomere restriction fragment (TRF) analysis (37) and telomere fluorescence *in situ* hybridization for ultra-bright telomeric foci (UTF). UTF was combined with immunofluorescence (IF) of the PML protein, which was discovered to co-localize with telomeres in ALT samples, to detect APBs, yielding an additional ALT feature (38). Recently C-circles, circular self-primed telomeric DNA repeats, have been shown to be a sensitive and specific biomarker for ALT in tumors (32) that also circulate in patient plasma, potentially increasing the clinical utility of C-circles as a biomarker (39, 40).

**Figure 1:**
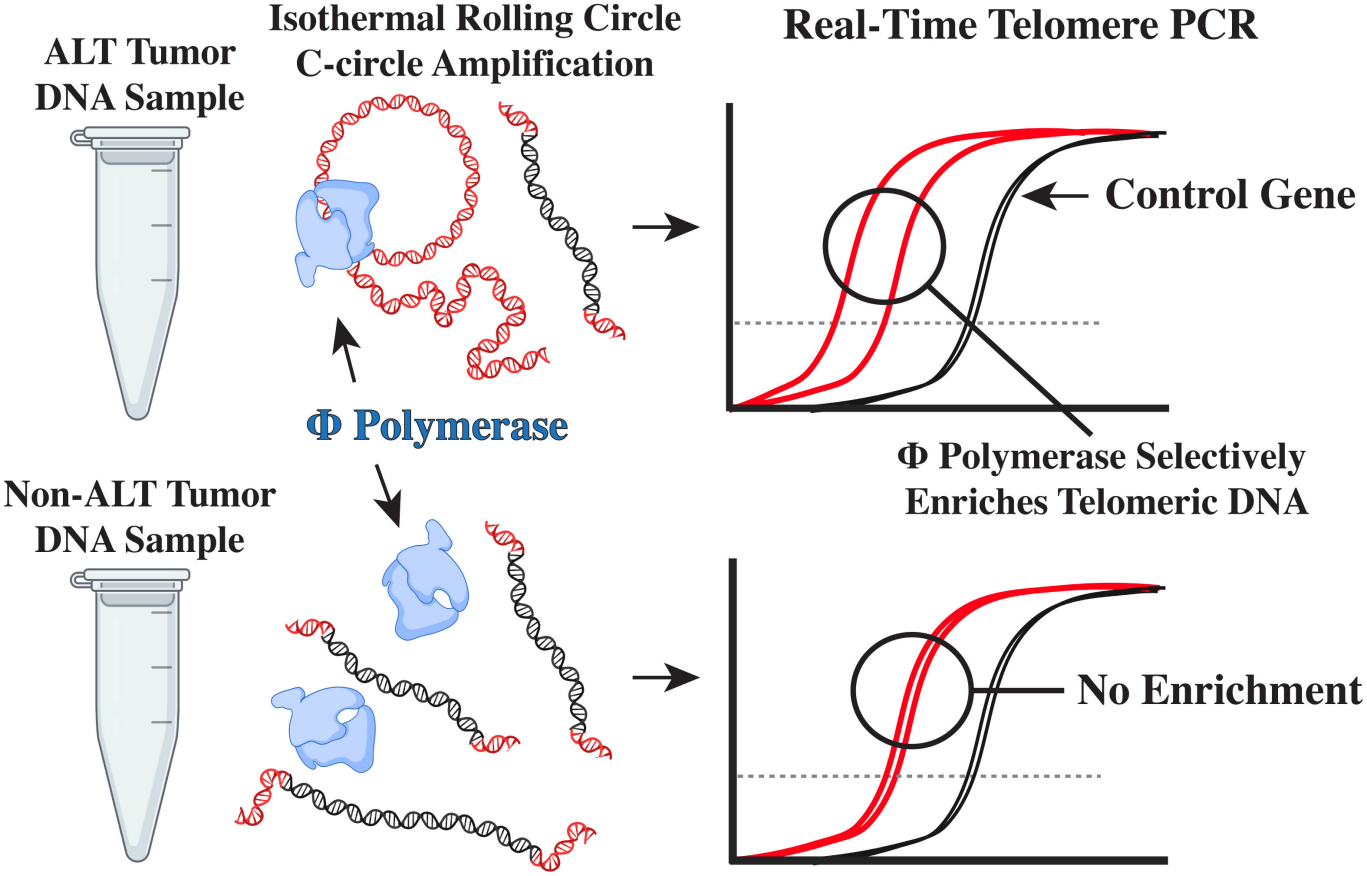
The real-time PCR CCA. Self-primed telomeric C-circles are selectively amplified by ϕ-29 polymerase via rolling circle amplification. Subsequent real-time PCR detection of telomere content reveals an enriched telomeric signal, indicating the presence of C-circles.

After genomic DNA is extracted (**Figure 1**) from fresh frozen tumor, or plasma, C-circles can be enriched and subsequently detected by blot or real-time PCR (41, 42). First, C-circles are selectively amplified by ϕ-29 DNA polymerase via isothermal rolling-circle amplification (**Figure 1**) (41, 43, 44), which enriches the partially double-stranded telomeric DNA, termed C-circles, when compared to a reaction without ϕ-29. Subsequently, the telomeric signals can be compared by real-time PCR (**Figure 1**) for the ϕ-29 and no ϕ-29 reactions (**Supplementary Figures S1A, C**), which is then normalized to a single copy gene (e.g. VAV2) for the same ϕ-29 and no ϕ-29 reactions (**Supplementary Figures S1B, D**) (41, 42). This unique molecular diagnostic assay allows for high-throughput screening of DNA from fresh frozen tumor and plasma samples with as little as 1 ng of template input (14, 41). Herein, we sought to assess the frequency of ALT in pediatric sarcomas using the real-time PCR CCA on DNA samples extracted from fresh frozen tumor.

## 2 Materials and methods

### 2.1 Tumor samples

Genomic DNA was extracted using the QIAamp DNA mini kit (Qiagen, 51104), in accordance with the manufacturer’s instructions, and stored at -20°C, or in liquid nitrogen vapor, until aliquoted and sent to TTUHSC on dry ice for use in the C-circle assay. *PAX3-FOXO1* or *PAX7-FOXO1* fusion status was previously determined on all samples without unambiguous embryonal RMS histology. Fusion status was determined by reverse transcriptase-polymerase chain reaction assays (45) of RNA isolated using RNA STAT-60 (Tel-Test, Friendswood, TX).

### 2.2 DNA quantification

Fluorometric quantification of DNA samples was carried out on a Qubit 2.0 system with the Qubit dsDNA Broad Range Assay Kit (Invitrogen Cat. No. Q32853).

### 2.3 The real-time PCR C-circle assay

The isothermal rolling circle amplification reactions were performed on an Eppendorf Vapo.Protect thermocycler at 30°C for 8 hrs, 65°C for 20 min, and held at 4°C. Reactions were comprised of: 32 ng of template DNA, 2 µL BSA (2 µg/µL), 2 µL of 1% Tween, 0.8 µL DTT (100µM), 2 µL of 10 mM dNTPs (NEB, Ipswich, MA, N0447L), 2 µL of ϕ-29 Buffer, 0.8 µL of ϕ-29 DNA polymerase (NEB, Ipswich, MA, M0269L), and nuclease-free water up to 20 µL. No ϕ-29 control reactions consisted of the aforementioned reagents with nuclease-free water in place of ϕ-29 DNA polymerase. After isothermal rolling circle amplification, all reactions were diluted with 20 µL nuclease-free water to a final volume of 40 µL.

Subsequent real-time PCR amplification of telomere DNA (Forward Primer: 5’ - CGGTTTGTTTGGGTTTGGGTTTGGGTTTGGGTTTGGGTT - 3’, Reverse Primer: 5’ - GGCTTGCCTTACCCTTACCCTTACCCTTACCCTTACCCT - 3’) and VAV2 DNA (Forward Primer: 5’ - TGGGCATGACTGAAGATGAC - 3’, Reverse Primer: 5’ - ATCTGCCCTCACCTTCTCAA - 3’) (IDT, Coralville, IA) was performed using a 96-well Thermo-Fisher Quantstudio 3 Real-Time PCR System with the following cycling conditions: Telomere reaction: 95°C for 15 min, 33 cycles of 95°C for 15 sec and 56°C for 2 min, and VAV2 Reaction: 95°C for 15 min, 40 cycles of 95°C for 15 sec, 57°C for 30 sec, and 72°C for 1 min. Real-time PCR reactions consisted of: 5 µL of diluted isothermal reaction product, 12.5 µL QuantiTect SYBR Green PCR Master Mix (Qiagen, 204445), 1 µL DTT (100 µM), 0.5 µL DMSO, 1 µL nuclease-free water, and 2.5 µL of primers (5 µM Tel, or 2 µM VAV2). All real-time reactions (Telomere ϕ, Telomere No-ϕ, VAV2 ϕ, VAV2 No-ϕ) were carried out in triplicate and assessed via arbitrary unit (AU) calculations. DNA from CHLA-90 and CHLA-20 cell lines were used for positive and negative controls, respectively. Samples were considered CC+ if they had ≥5 AU, after normalization to CHLA-90, as previously described (46–48).

### 2.4 Statistical analysis

The relationship between clinical characteristics and C-circle status (**Table 4**) was evaluated by Chi-square, or Fisher’s exact test, when appropriate. The Mann-Whitney U Test was used to analyze telomere content. Two-tailed statistical tests with *P* values ≤ 0.05 were considered significant. All analyses were performed in GraphPad Prism v10.2.2.

**Table 4:** Clinicopatholgical data for the osteosarcoma sample cohort.

Osteosarcoma		C-circle Positive	C-circle Negative	P Value
Sex				
Male		17	11	0.16
Female		26	7	
NA		14	9	
Age				
< 18 years		40	17	0.83
> 18 years		6	3	
NA		11	7	
Location				
Axial		5	2	0.96
Extremity		34	13	
Metastasis		5	3	
NA		13	9	
Histology				
Chondroblastic		1	0	N/A
Fibroblastic		1	1	
Osteoblastic		5	5	
Telangiectatic		1	0	
Osteoblastic & Chondroblastic		1	1	
Osteoblastic & Fibroblastic		1	0	
Osteoblastic & Sclerosing		1	0	
Osteoblastic & Telangiectatic		1	0	
Osteoblastic, Chondroblastic & Telangiectatic		1	0	
NA		44	20	
Response				
Responder		12	6	0.65
Non-responder		24	9	
NA		21	12	

NA, Not Applicable.

## 3 Results

### 3.1 Patient cohort

The Children’s Oncology Group (COG) Biopathology Center provided 82 RMS DNA samples from residual stored DNA. These RMS specimens were collected from patients enrolled on a variety of Intergroup Rhabdomyosarcoma Study Group or COG Soft Tissue Sarcoma studies and received as de-identified samples. All Ewing sarcoma cases were part of COG clinical trial AEWS0031. All cases were reviewed by COG pathologists and a EWS-ETS fusion gene was identified in all cases. None were Ewing-like tumors with *FET-ETS* or *CICX-DUX4*. All cases expressed an *EWS-FLI1* or *EWS-ERG* fusion gene and were part of the NCI Strategic Partnering to Evaluate Cancer Signatures (SPECS) program for childhood sarcoma gene expression profiling (49). EFS, OS, and additional RMS DNA was isolated from primary tumors obtained under informed consent by COG and processed by the pediatric division of Cooperative Human Tissue Network at Nationwide Children’s Hospital. These anonymized samples were originally used for genomic analyses in the NCI SPECS program, and in the case of OS, also the NCI TARGET program, and, in both cases, exempt from Human Subjects Research per IRB review (49–51).

### 3.2 CCA results

**Figure 2:**
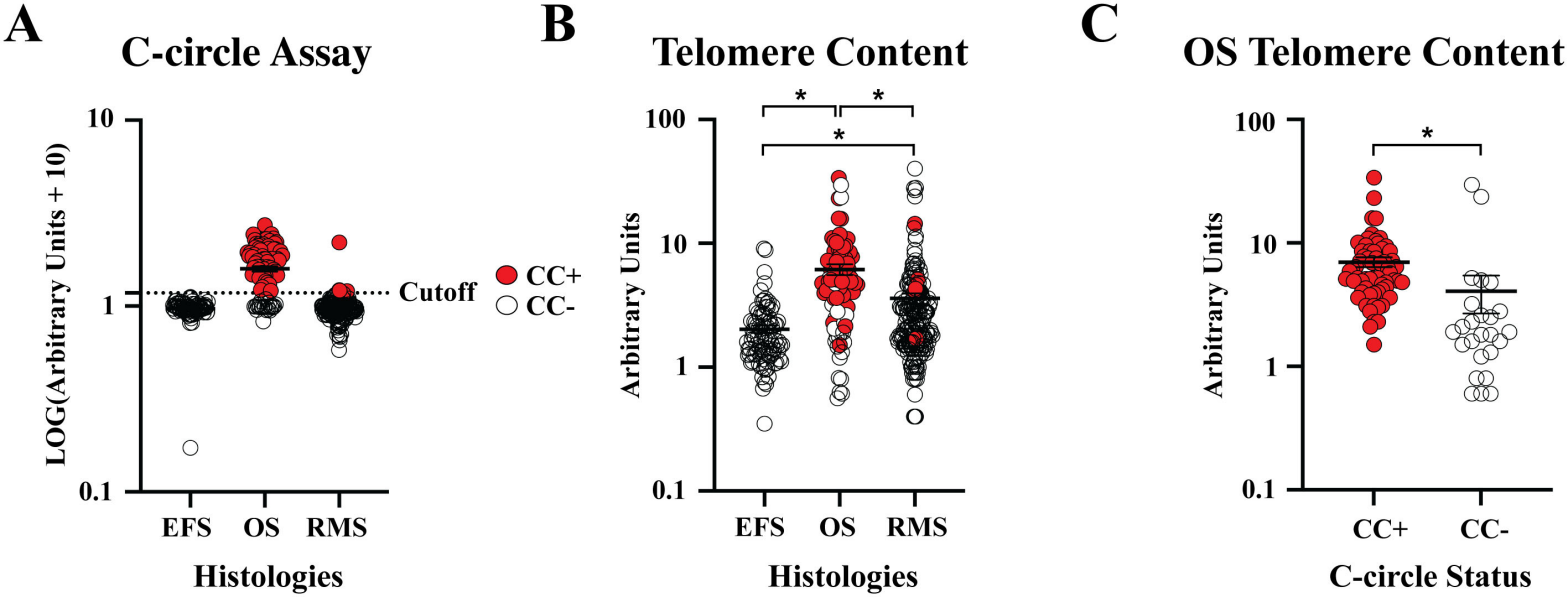
Patient sample CC status and telomere content. **(A)** Normalized relative CC content was plotted by tumor histology. Samples above the previously established cutoff of 5 arbitrary units (AU) were considered CC+. **(B)** Telomere content, normalized to CHLA-90 at 5 AU, were plotted by histology. **(C)** Telomere content was plotted for CC+ and CC- OS samples. * P < 0.05.

Of the 373 DNA samples received (n = 95 EFS, n = 191 RMS, and n = 87 OS), five samples (1 EFS and 4 RMS) did not amplify due to poor DNA quality. CCA results are shown in **Figure 2A**. We observed 0 of 94 (0%) CC+ EFS cases, which is in concordance with previous reports (**Table 1**) (10, 12, 23). In contrast to EFS, 62 of 87 (71%) of OS tumors were CC+, which fell within the range of prior studies (12, 23). We did not observe statistically significant relationships between C-circle status and the clinicopathological data (**Table 4**), which aligns with the conclusions of previous studies that identified ALT through methods other than the CCA (14–16). We observed 5 of 187 (2.7%) CC+ RMS in the sample cohort (**Table 5**), which is lower than the previously reported 6%, which was determined by APB analysis (14). Of the 5 CC+ RMS samples identified, four were fusion negative (FN) embryonal RMS (ERMS) and one was fusion positive (FP) alveolar RMS (ARMS).

**Table 5:** PAX3/7-FOXO1 fusion status rhabdomyosarcoma sample cohort.

Rhabdomyosarcoma		C-circle Positive	C-circle Negative
*PAX3/7-FOXO1* Fusion Status			
Fusion Positive		1	87
Fusion Negative		4	74
NA		0	21

NA, Not Applicable.

### 3.3 Telomere content

Telomere content amongst EFS, OS, and RMS (**Figure 2B**) ranged from 0.35 - 9.1, 0.56 - 33.73, and 0.93 - 14.42, respectively. Each histology showed a significant difference (p < 0.05) in telomere content, and CC+ OS had a significantly higher (p < 0.05) telomere content than CC- OS samples (**Figure 2C**), which is in concordance with reports that ALT telomere content is generally higher than non-ALT samples (39, 52).

## 4 Discussion

The prognostic value of ALT, and other TMM, is gaining traction (23, 33, 53), including in veterinary care (54). Studies have linked high telomerase expression with exceptionally aggressive tumors that can result in rapid progression and poor clinical outcomes (15, 33). By contrast, ALT has been associated with indolent disease progression; yet, patients with various tumor types have been observed to have a worse overall survival (23, 28, 33, 55). Recently, we observed high amounts of ATM kinase activation (which promotes chemotherapy resistance) in patient-derived neuroblastoma cell lines (PDCLs) and patient-derived xenografts (PDXs) (48), and also in PDCLs of other histologies (rhabdomyosarcoma, osteogenic sarcoma, triple negative breast cancer, and colorectal cancer) that have the ALT phenotype (47). We have also observed that certain clinical stage drugs (an ATM kinase inhibitor (48) and a p53 reactivator (47)) are active in reversing chemotherapy resistance in ALT PDCLs and PDXs. Thus, robust identification of ALT has the potential to be a prognostic biomarker and a companion diagnostic for ALT-targeted therapies.

Generally, ALT is activated by loss-of-function (LOF) genetic alterations in the chromatin remodelers α thalassemia-mental retardation, X linked (*ATRX*) (33) and death domain-associated protein 6 (*DAXX*) (56). *ATRX* inactivating mutations are commonly observed among different tumor types, while *DAXX* mutations are primarily associated with pancreatic neuroendocrine tumors (PanNETs) (56). ALT is less frequently associated with LOF alterations in *H3F3A *(57, 58) and *SMARCAL1* mutated tumors (59, 60). Previous studies have used these genomic alterations as proxies to identify ALT, but depending on histology, as many as ½ ALT cancers can be wild-type for *ATRX* or *DAXX* (41, 61).

C-circles, *TERT* expression, high telomere content with heterogenous telomere length, and APBs have been used to screen sample sets to establish ALT frequencies amongst sarcomas; however, each of these techniques have their own advantages and disadvantages. Relatively fragile, C-circles can be degraded by excess freeze-thaw cycles, prolonged vortexing, and formalin-fixing; thus, proper sample handling and storage are required (62). Recently, ALT tumors have been shown to protect C-circles from nuclease degradation in the blood by releasing C-circles within exosomes, which may provide a non-invasive blood-based biomarker for the detection and monitoring of ALT tumors *in vivo* (40). Although there is no standardized method for determining ALT status (28), C-circles are the only known molecule specific to ALT (40), and the molecularly based real-time PCR C-circle assay can utilize DNA that has been isolated for sequencing; thus, it is readily translatable to the clinical laboratory, and it’s for these reasons that we selected this approach (28, 32, 42, 61).

We observed no CC+ EFS cases, which is likely due to the activation of *TERT* by EFS fusion proteins (39). The ALT phenotype is known to be essentially exclusive to *TERT* activation (32, 39, 40). In OS patients, expression of *TERT* has been shown to portend an unfavorable clinical prognosis (15); however, stage and clinical outcomes of ALT cases were shown to be equivalent to TA cases (16), but, the ALT phenotype provides a potentially targetable mechanism present in the majority of OS patients, some of which have poor clinical outcomes (47, 63, 64).

ALT is also known to occur in RMS (14), the most common pediatric soft tissue sarcoma (65). Classically, pediatric RMS cases were generally categorized histologically as ERMS, which was linked with better prognoses, or ARMS, which was associated with poor clinical outcomes (66). Further, molecular identification of *PAX3*, or *PAX7*, fusions with forkhead box protein O1 (*FOXO1*), is currently considered the preferred method of distinguishing the latter from the former (67). Instead of histologic criteria, which are inexact, the fusion status identifies ARMS and ERMS, which are FP and FN, respectivly (68).

The tested RMS samples were from banked DNA extracted from fresh frozen tissue; thus, it is possible that the age of the samples, or excess freeze-thaw cycles could have contributed to the lower ALT frequency, due to the degradation of C-circles (41). APB analysis from a previous study (14) has the advantage of using FFPE material, which enables distinguishing of tumor cells from stromal tissue; however, the APB assay is very labor intensive, not all ALT samples have APBs (33), and C-circles have been postulated to be more specific than other ALT markers (32). Ideally, future studies should evaluate the various methods for detecting ALT in the same histology within the same patient sample cohort, since each ALT marker is not necessarily present in every ALT sample or tumor model (14, 33). However, our data suggests that the real-time PCR CCA can identify ALT in sarcomas, and it has potential as a companion diagnostic assay for ALT targeted therapies in RMS, and especially OS, patient populations.

## Data Availability

The original contributions presented in the study are included in the article/**Supplementary Material**. Further inquiries can be directed to the corresponding author.
