# Supplementary material for: Prevalence of alternative lengthening of telomeres in pediatric sarcomas determined by the telomeric DNA C-circle assay
Source: Front Oncol. 2024 Aug 19;14:1399442. doi: 10.3389/fonc.2024.1399442 (PMC11366626; doi:10.3389/fonc.2024.1399442)
Supplement: Supplementary file 1 [file DataSheet1.docx]

Supplementary Material

# Supplementary Figure


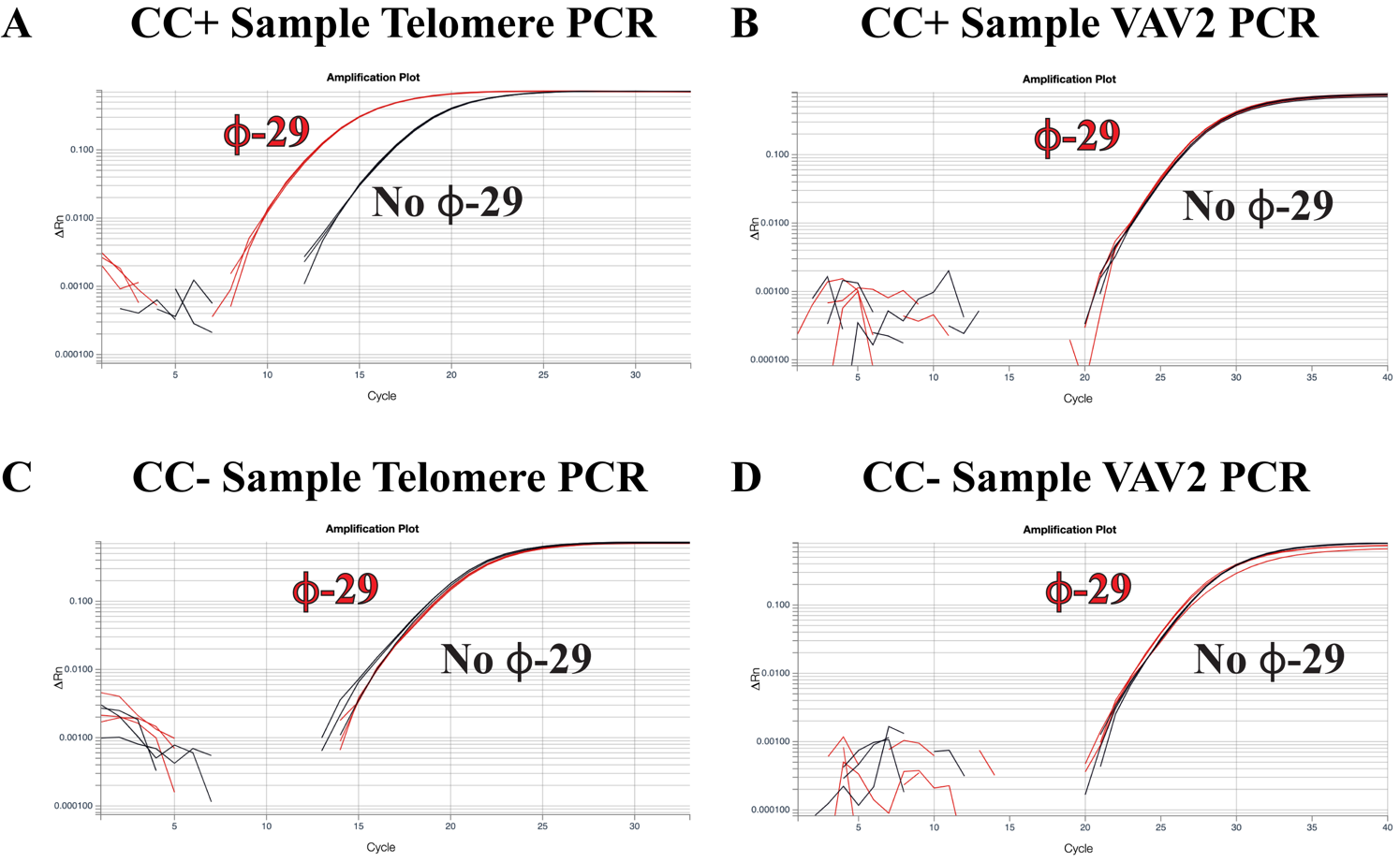


**Supplementary Figure 1.** **Representative** **Real-time PCR CCA Run Data for a C Circle (CC)+ and a CC- Patient Sample.** **(A)** Telomeric amplification of the φ-29 (C_q_ = 11.3) and no φ-29 (C_q_ = 16.3) reactions for a CC+ sample (n = 3). **(B)** Single copy control gene (VAV2) amplification of the φ-29 and no φ-29 reactions for a CC+ sample (n = 3). **(C)** Telomeric amplification of the φ-29 and no φ-29 reactions for a CC- sample (n = 3). **(D)** Single copy control gene (VAV2) amplification of the φ-29 and no φ-29 reactions for a CC- sample (n = 3).
